# Vascular Endocrine-Disrupting Effects of Bisphenol F and Bisphenol S on Human Umbilical Artery

**DOI:** 10.3390/jox16030111

**Published:** 2026-06-13

**Authors:** Fatima Abrantes-Soares, Mariana Marques Santos, Melissa Mariana, Margarida Lorigo, Elisa Cairrao

**Affiliations:** 1 RISE-Health, Department of Medical Sciences, Faculty of Health Sciences, University of Beira Interior, Av. Infante D. Henrique, 6200-506 Covilhã, Portugal; fatima.soares@ubi.pt (F.A.-S.); mariana.m.santos@ubi.pt (M.M.S.); melissa.r.mariana@gmail.com (M.M.); margarida.lorigo@gmail.com (M.L.); 2 FCS-UBI, Faculty of Health Sciences, University of Beira Interior, Av. Infante D. Henrique, 6200-506 Covilhã, Portugal

**Keywords:** BPA substitutes, fetal–maternal exposome, calcium channels, cGMP signaling pathway, plasticizers

## Abstract

In recent years, bisphenol F (BPF) and bisphenol S (BPS) have been used in several everyday products to replace bisphenol A (BPA), since exposure to BPA has been associated with the development of several pathologies. However, recent studies have also been associating exposure to BPA substitutes with the development of various pathologies, including cardiovascular diseases, and the safety of BPA substitutes for human health has been questioned. Thus, this study aimed to investigate and compare BPA, BPF and BPS effects on arterial tone and to explore the mechanisms involved. The results suggest that BPA, BPS and BPF exert non-genomic and endothelium-independent relaxant effects on arteries and smooth muscle cells from the umbilical cord. Regarding genomic effects, the results suggest that BPA, BPF, and BPS disrupted the primary mechanisms underlying HUA relaxation by interfering with the cGMP signaling pathway and modulating the Ca^2+^ channels activity. Moreover, these results suggest that BPF alters the vasorelaxant response more than BPA and BPS. Therefore, replacing BPA with its substitutes does not appear to be beneficial for human cardiovascular health. Thus, in the future, the vascular effects of these bisphenols should be further evaluated to clarify their modes of action and future implications for maternal-fetal health.

## 1. Introduction

Endocrine-disrupting chemicals (EDCs) have been defined by the Endocrine Society as exogenous chemical substances, or mixtures of chemical substances, that can interfere with any aspect of hormonal action [1,2]. In fact, EDCs are chemical compounds of both natural and synthetic origin, present in a variety of everyday products, including plastic packaging, toys, cosmetics, detergents, flame retardants and pesticides [3,4]. These compounds are also widely used in coating canned products and in food storage and can release monomers into food or drink, which are absorbed by the body and disrupt the endocrine system [5,6].

The EDCs can influence hormone signaling pathways through various mechanisms. They can mimic hormones such as estrogens, androgens and thyroid hormones, act as antagonists by binding to intracellular endogenous hormone receptors, preventing the binding of natural hormones, and interfere with or block the action of hormones or their receptors [7,8]. Therefore, exposure to these compounds has been associated with the development of several pathologies or morphological alterations, particularly during vulnerable periods of life, such as pregnancy and the prenatal phase [9]. Currently, bisphenols are regarded as among the most relevant EDCs due to their high production volumes and ubiquitous presence [4].

Specifically, bisphenol A (4-[2-(4-hydroxyphenyl)propan-2-yl]phenol; BPA) is a phenolic compound widely used in numerous industries due to its lightweight, durability and heat resistance, and it serves as the primary raw material for epoxy resins and polycarbonate plastics [10,11]. As the need to replace and eliminate BPA from the market grows, industries are turning to structurally similar monomers whose effects on human health are less well understood, namely bisphenol F (4-[(4-hydroxyphenyl)methyl]phenol; BPF) and bisphenol S (4-(4-hydroxyphenyl)sulfonylphenol; BPS) [12,13,14]. Regarding their chemical properties, BPF enhances the durability and thickness of epoxy resins and polycarbonates [15,16], though its high solubility leads to frequent detection in aquatic environments [17]. Conversely, BPS offers superior thermal and UV stability, reducing leaching from consumer products [18,19]; though this resilience makes it more resistant to biodegradation, facilitating its environmental persistence and accumulation [18]. Despite these ecological drawbacks, BPS is increasingly adopted in the manufacture of plastics, resins, and thermal papers [15,18,20].

Indeed, some investigations have associated exposure to BPA substitutes with the development of various pathologies, such as reproductive [21,22], neurological [23], thyroid [24,25], behavioral [26,27], immunological [28,29], and cardiovascular [30]. Furthermore, several studies reported the presence of BPA substitutes in different human biological samples, even during the pre- and post-natal periods, namely, maternal blood [31], urine [32], umbilical cord and placenta [33], amniotic fluid [34] and breast milk [35]. Worryingly, some studies conducted in animal models and humans have reported that BPA substitutes can be transferred from mother to fetus across several generations, impairing fetal health [33,36,37,38]. However, the adverse outcomes associated with exposure to BPA substitutes remain unclear at the human cardiovascular level.

Therefore, the present study aimed to investigate how exposure to BPA substitutes—BPF and BPS—can impair vascular homeostasis in umbilical cords of pregnant women without pathologies, comparing their effects with those of BPA. Given their ease of isolation from the human umbilical cord (HUC) and for being an excellent model for studying the vascular effects and implications of EDCs on the vascular system of pregnant women, the human umbilical artery (HUA) was used in this study [4,39]. Furthermore, human umbilical artery smooth muscle cells (HUASMCs) can be isolated from HUA, and the regulation of their contractile mechanisms is essential for the control of fetal-placental blood flow. Therefore, HUA rings without endothelium were used to investigate the non-genomic and genomic effects of these bisphenols on vascular contractility by ex vivo organ bath experiments, while HUASMC were used to evaluate the effects of these EDCs at the cellular level, performing in vitro experiments of Planar Cell Surface Area (PCSA) and cell viability assay (MTT).

## 2. Materials and Methods

### 2.1. Ethics Statements

For this study, all biological samples were obtained from the obstetrics unit of Unidade Local de Saúde Cova da Beira (ULSCBeira; Covilhã, Portugal). All procedures conducted with these samples were approved by the Ethics Committee of the Unidade Local de Saúde Cova da Beira (No. 33/2018, 18 July 2018) and are consistent with the principles of the Declaration of Helsinki. The collection of the HUC samples and the obtaining of informed consent from the donor mothers residing in an inland region of Portugal (Beira Interior) were performed by professionals (medical staff, nurses, health technicians, and assistants) present in the hospital delivery room and are in compliance with the law on the protection of personal data in Portugal (Law No 67/98 of 26 October) and the European Union’s General Data Protection Regulation (GDPR, EU Regulation No 2016/679 of 27 April).

### 2.2. Sample Collection

A total of seventy-one HUC samples were collected from pregnancies without pathology after vaginal delivery. Donor mothers who were taking medication other than folic acid during the first 21 weeks of gestation and iron supplements or other vitamins during the entire gestation period were excluded from the study. The HUC samples were cut from the proximal half of the newborn (20 cm) and collected within the first few minutes after delivery. These samples were stored in a sterile physiological saline solution (PSS; pH 7.4; composition: 110 mM NaCl, 0.15 mM CaCl_2_, 5 mM KCl, 2 mM MgCl_2_, 10 mM HEPES, 10 mM NaHCO_3_, 0.5 mM KH_2_PO_4_, 0.5 mM NaH_2_PO_4_, 10 mM Glucose and 0.49 mM EDTA), to which a mixture of antibiotics (penicillin (5 U/mL), streptomycin (5 µg/mL) and amphotericin B (12.5 ng/mL)) was added in order to avoid contamination. Finally, HUC samples were stored at 4 °C for 4–24 h. All experiments were performed using several HUAs from at least three different HUCs to ensure genetic variability. It is important to note that the majority of the umbilical cords collected were used for ex vivo studies and cell culture to be used in cell contractility and cell viability studies.

### 2.3. Tissue Preparation

To perform arterial contractility experiments, HUA was isolated from HUC pieces. All experiments were performed with endothelium-denuded HUA to determine the effects of BPA, BPF, and BPS on arterial smooth muscle contractility and to exclude endothelium-induced relaxation. Tissue preparation was performed as described by Fonseca et al. [10]. Briefly, HUAs were isolated from HUC pieces by removing Wharton’s jelly (the surrounding connective tissue). Then, HUAs were cut into 3–5 mm rings, and the vascular endothelium was mechanically removed, gently introducing a needle with cotton thread through the arterial lumen.

### 2.4. Contractility Experiments in HUA Rings

The organ bath technique is an ex vivo method used to evaluate the direct (non-genomic) effects and the effects after 24 h of exposure (genomic) to BPA, BPF and BPS in HUA. The artery tension recordings were performed according to our previous works [10,39,40]. Different HUAs (*n*) were used for each independent experiment.

Briefly, HUA rings were suspended between two parallel stainless-steel wires and placed into the organ bath chamber to measure the isometric tension (millinewton, mN). Throughout the experience, these rings were continuously carbonated (95% O_2_ and 5% CO_2_). Initially, HUA rings were subjected to resting tension (basal, 20–25 mN) and exposed to a 45 min equilibration period. After this time, the viability of the HUA rings was tested by challenging them to a supramaximal concentration of serotonin (5-HT, 1 µM). For the following procedures, only rings with maximum contraction exceeding 10 mN were used in this study.

Firstly, the direct effects of BPA, BPF and BPS (0.002, 0.02, 0.2, 2, 20 and 100 µM) were analyzed. The bisphenol concentrations were selected based on previous studies, either conducted by our research group and others, using rat smooth muscle cells (SMCs) [41] and cat uterine SMCs [42], human umbilical SMCs [10] and vein endothelial cells (HUVECs) [43] and human cardiac AC-16 cells [44]. In addition, the in vitro–in vivo scaling factor, stating that to cause similar biological effects, the in vitro concentrations have to be 20–200-fold higher than the maximum concentration observed in the human plasma, was also considered [4,45]. Therefore, HUA rings were contracted with 5-HT (1 µM) or potassium chloride (KCl, 60 mM). These two agonists enable the assessment and differentiation of different contraction mechanisms, with one being receptor-dependent (5-HT) and the other receptor-independent (KCl). Specifically, 5-HT is a potent vasoactive agent whose contractile response occurs through inhibition of adenyl cyclase (5-HT_1B_/5-HT_1D_) and stimulation of the PLC/IP3 pathway (5-HT_2A_), increasing intracellular Ca^2+^ concentration, while KCl acts through depolarization, increasing Ca^2+^ influx through voltage-gated channels [46]. Furthermore, 5-HT was chosen as a vasoactive mediator because its significance in cardiovascular pathologies in pregnant women has already been demonstrated, particularly regarding changes in concentration levels in pre-eclampsia [4,47]. After stabilization of the contractile response induced by these agents, different concentrations of BPA, BPF and BPS (0.002–100 µM) were added. Ethanol (vehicle) was used to perform control experiments at the same percentage used to dissolve the bisphenols, and its concentration never exceeded 0.1%.

Secondly, the genomic effects of bisphenols on HUA were analyzed, and mechanistic studies were performed to clarify the mechanisms of action of these EDCs. Therefore, HUA rings were incubated in Dulbecco’s modified Eagle’s medium F12 (DMEM-F12) for 24 h in the presence of BPA, BPF or BPS (0.002, 0.2 and 20 µM). Subsequently, these rings were contracted with the contractile agents, 5-HT (1 µM) or KCl (60 mM), and a stable contraction was achieved. To determine the involvement of cyclic nucleotides and Ca^2+^ channels in bisphenol-induced vasorelaxation, HUA rings were submitted, respectively, to sodium nitroprusside (SNP, 0.01; 0.1; 1; 10 and 100 µM)—a stimulator of soluble guanylate cyclase (sGC)—and nifedipine (Nif, 0.01; 0.1 and 1 µM)—an inhibitor of L-type calcium channels (LTCC). These signaling pathways are considered the main ones involved in HUA-induced smooth muscle relaxation, which is why they were selected for this study [48]. Since SNP and Nif are photodegradable agents, all the experiments were performed in the absence of light.

### 2.5. Cell Culture and Dissociation

The HUASMCs were isolated from HUA, as previously described by Lorigo et al. [39]. Briefly, HUA were isolated from HUC pieces by removing Wharton’s jelly. Subsequently, HUA were cut into small rings, and the tunica intima was mechanically removed by gentle rubbing with a cotton bud, to avoid contamination with endothelial cells. Afterwards, the smooth muscle layers from the tunica media were extracted and placed in a small Petri dish containing 3 mL of PSS and 30 µL of antibiotic. These layers were then transferred to cell culture flasks (T25), previously coated with collagen (5 µg/cm^2^) to promote adhesion. The layers were kept in culture at 37 °C under an atmosphere of 95% air and 5% CO_2_ in 4 mL of HUASMC-specific culture medium (pH = 7.40; composition: DMEM-F12, bovine serum albumin (BSA, 0.25%), epidermal growth factor (EGF, 5 μg/mL), fibroblast growth factor (FGF, 0.05 μg/mL), heparin (2 μg/mL), fetal bovine serum (FBS, 5%), mixture of antibiotics and insulin (5 μg/mL)). The culture medium was renewed every 2 to 3 days, and after 25 to 35 days, confluent primary cultures were obtained. Subcultures of HUASMC were obtained up to a maximum of 5 passages, and cells from different passages were used to perform the cell contractility (PCSA) and cell viability (MTT) experiments.

### 2.6. Contractility Experiments in HUASMC

The Planar Cell Surface Area (PCSA) technique was used to study the cellular contractility of HUASMCs, as previously described by Lorigo et al. [48]. In this technique, images of the HUASMCs are recorded over time, allowing the study of changes in cell surface area. By analyzing an increase or decrease in the cell area, it is possible to verify whether the cells have relaxed or contracted, respectively [48]. For each experiment, SMC from at least 3 HUAs of different umbilical cords were used.

Briefly, the HUASMCs were placed in a 6-well plate, and when they reached confluence, they were cultured in FBS-free culture medium (composition: DMEM-F12, BSA (0.25%), and antibiotic solution) for 24 h. To study the genomic effects of these compounds, the HUASMCs were incubated for a further 24 h with BPA, BPF, and BPS (20 µM) under ambient conditions. After these 48 h, the cells were trypsinized into specific Petri dishes, previously coated with collagen (5 µg/cm^2^) and kept in the dark for 2 h. The medium without FBS was then removed, and the HUASMCs were washed four times with 500 µL of modified PCSA solution (composition: 124 mM NaCl, 5 mM HEPES, 10 mM TEA, 6 mM Glucose, 5 mM CaCl_2_ and 4.7 mM KCl). The cells were observed using an inverted fluorescence microscope (Zeiss Axio Observer Z1, Jena, Germany) equipped with a high-speed monochrome digital camera (Axio Cam Hsm, Zeiss, Jena, Germany). It also incorporates an incubation system with temperature control, which is crucial for maintaining cell viability throughout the experimental procedure. Photographs were taken of the cells at 20-minute intervals throughout each experiment, before and after the addition of the different compounds. This time interval corresponds to the time required to reach a maximum response to the added agent’s action.

To study the direct effects, HUASMCs were contracted with 5-HT (1 µM), and after the cell contraction reached its maximum response, different concentrations of BPA, BPF, and BPS (2 and 20 µM) were added. Control experiments were always performed with ethanol (0.02%). To study the genomic effects of bisphenols, BPs-incubated cells were contracted with 5-HT (1 µM), and after cell contraction reached its maximum response, concentrations of SNP (10 µM) and Nif (1 µM) were added.

Finally, to analyze the results and perform the image processing, computer programs were used, namely, the Automatic measurement supplement of the Axionvision 4.8 software (Zeiss, Jena, Germany). In this analysis, cell area was measured using the supplementary ‘‘Automatic Measurement program’’ (Zeiss, Jena, Germany). Every experiment used a set of cells from only a single culture and passage. To ensure variability in the data, the same experimental protocol was repeated in cells from different passages and different cultures.

### 2.7. Cytotoxicity Assays (MTT)

The cell viability of HUASMC (from 5 HUAs, each from a different umbilical cord, n = 5) was assessed in response to BPA, BPF, and BPS exposure using the MTT assay, following the methodology outlined by Gloria et al. [40]. Briefly, once the cells reached confluence in 96-well plates, they were incubated with different concentrations of BPA, BPF and BPS (0.0002, 0.002, 0.02, 0.2, 2, 20, 100, 200 and 1000 µM) and ethanol (0.2% and 1%). In addition to the reasons mentioned above, the selection of this wide range of concentrations is also related to the endocrine-disrupting properties of bisphenols, as they do not exhibit a typical monotonic dose–response curve [49]; that is, an increase in dose does not correspond to a greater effect, and vice versa. After a 24 h incubation period, in the absence of light, the culture medium containing the compounds was removed, and 100 µL of MTT (0.5 mg/mL) was added to each well. The HUASMC were incubated for 3 h and 30 min at 37 °C, in an atmosphere of 95% O_2_ and 5% CO_2_. After this period, the MTT was removed and replaced with 100 µL of dimethylsulphoxide (DMSO) to dissolve the formazan crystals that had formed. The amount of formazan produced was measured at 570 nm and 690 nm using a photometer (EZ Read 400, Microplate Reader, Biochrom, Lisboa, Portugal).

### 2.8. Gene Expression Assays (qPCR)

Total RNA was extracted from HUASMC pre-incubated (24 h) with BPA, BPF or BPS at concentrations of 0.002, 0.02, 0.2, 2 and 20 µM. In all the assays, a solvent control (ethanol 0.02%) was also included, corresponding to the maximum concentration of BPs dilution. The RNA extraction (20 µL) was performed using the TripleXtractor reagent (GRiSP Lda, Porto, Portugal) and following the manufacturer’s instructions. The isolated RNA was evaluated by spectrophotometry (260/280 nm ratio) (NanoDrop One (Thermo Scientific, Lisboa, Portugal)), considering absorbance values between 1.8 and 2.0. Moreover, the RNA integrity was confirmed by the presence of two sharp bands (28S and 18S) in a 1% agarose gel electrophoresis, stained with GreenSafe Premium (MB13201, NZYTech, Lisboa, Portugal), and visualized under UV light on a transilluminator (UVITEC Cambridge, UK, FireReader v15.15). The cDNA synthesis (BIO-RAD T100 TH Thermal Cycler, BioRad, Hercules, CA, USA) was performed using the NZY M-MuLV Reverse Transcriptase (MB08301, 20,000 U, NZYTech, Portugal), following the manufacturer’s recommendations, namely 1 µg of total RNA/per reaction, joined to a random hexamer mix (MB12901, NZYTech, Portugal) and dNPTs NZYMix (MB08601, 25 mM each, NZYTech, Portugal) at 65 °C, 5 min (denaturation step). Following a cool on ice, the enzyme mix (including 10× reaction buffer and reverse transcriptase) was added to a final volume of 20 μL per reaction. The process is continued by an incubation at 25 °C for 10 min to facilitate random primer binding, followed by 37 °C for 50 min. The final step is the enzyme inactivation at 70 °C for 15 min. Following cDNA synthesis, two genes (*sGC*, GenBank ID: XM_034935167.1, and *Cav1.2*, GenBank ID: NM_001379671.1) [39] involved in cardiovascular functions were selected for expression analysis by quantitative real-time PCR (CFX Connect TM Real-Time System, BioRad, Hercules, CA, USA). The PCR reactions (20 µL, Bio-Rad CFX Manager v3.1 software) were run using NZYSpeedy qPCR Green Master Mix (2×) (MB22402, NZYTech, Portugal) with synthesized cDNA (1 µL) and primers (300 nmol/L). The amplification comprises a denaturation step at 95 °C for 5 min, followed by 40 cycles at 95 °C (10 s each), an annealing cycle at 60 °C (30 s) and a final extension at 72 °C for 20 s. Melting curves (55 °C to 95 °C at an increment of 0.5 °C for 0.05 s) were also performed. Relative gene expression was calculated by the 2^−ΔΔCT^ mathematical model [50].

### 2.9. Drugs and Chemicals

All drugs and chemical compounds used were purchased from Sigma-Aldrich Química (Sintra, Portugal). Stock solutions of BPA, BPF, BPS and Nif were dissolved in pure ethanol, while 5-HT and SNP were dissolved in Milli-Q water. All these solutions were stored at −20 °C. Dilutions of these compounds were prepared for various tests: in the arterial contractility test, dilutions were made in Krebs solution; in the cell contractility tests, they were made in modified PCSA solution and culture medium without FBS; in the MTT tests, they were prepared in culture medium. These dilutions were performed daily according to the requirements of each experiment.

### 2.10. Statistical Analysis

The statistical data was graphed using Origin 2021 9.8.0.200 software. The results are presented as the mean ± standard error (S.E.) of n independent experiments. Statistical analysis was conducted using the SigmaPlot Statistical Analysis System program, version 15.0 (2022). Different statistical methods were selected based on the number and type of variables tested. Statistically significant differences were considered when the probability level was less than 5% (*p* < 0.05).

For the arterial contractility studies, two-way ANOVA followed by Holm–Sidak post hoc tests were used to evaluate the effects of BPA, BPF and BPS on HUA pre-incubated and contracted with 5-HT and KCl, as well as to assess the effects on SNP and Nif activity. In the cell contractility experiments, one-way ANOVA, followed by Dunn’s and Holm–Sidak’s post hoc tests, was used to examine the effects of BPA, BPF, and BPS on HUASMCs pre-incubated in a contracted state with 5-HT, in addition to the effects of SNP and Nif. For the cell viability experiments, one-way ANOVA was also conducted, followed by Dunn’s post hoc tests. For the gene expression assays, one-way ANOVA was also conducted, followed by Dunn’s or Holm–Sidak post hoc tests. For all tests, the criteria of normality and the homoscedasticity of data were checked by Kolmogorov–Smirnov and Levene’s mean tests, respectively.

## 3. Results

To investigate the complex impact of BPA, BPS, and BPF on HUA and HUASMC contractility, this work characterized their rapid non-genomic actions alongside their genomic effects. The non-genomic pathway manifests as immediate cellular responses without prior incubation, whereas genomic effects require the modulation of downstream gene expression [10,39]. Consequently, a 24 h incubation window was implemented for both HUA and HUASMCs to evaluate these genomic alterations. This exposure period aligns with previous protocols [10,39,40], proving that 24 h provides ample time for genomic changes to occur. From a mechanistic perspective, these bisphenols disrupt gene expression networks following relatively brief exposure (24 h), resulting in the observed changes in arterial contractile dynamics.

### 3.1. Direct Vascular Effects of BPA, BPF and BPS on HUA

To analyze the direct effects of bisphenols, HUA rings without endothelium were contracted using a receptor agonist (5-HT) and depolarized with isosmotic KCl solution (60 mM).

**Figure 1:**
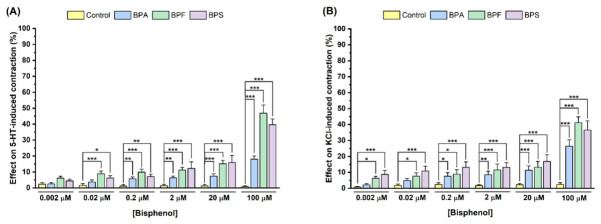
Effect of BPA, BPF and BPS (0.002 μM–100 μM) on human umbilical arteries (HUA) contracted with serotonin (5-HT; 1 μM) (**A**) and potassium chloride (KCl; 60 mM) (**B**). The results are expressed as a percentage (%) of relaxation obtained after contraction induced by 5-HT or KCl. The bars represent the mean value and the vertical lines the standard error (S.E.) of at least 5 independent experiments of different HUA (n = 5), with two to three replicates for each bisphenol’s concentration. Statistical analysis was carried out using a two-way ANOVA test, followed by the Holm–Sidak post hoc test, where * (*p* < 0.05), ** (*p* < 0.01), and *** (*p* < 0.001) represent statistically significant differences when compared to the control.

Figure 1A illustrates the direct effects of BPA, BPF and BPS (0.002–100 μM) on HUA rings without endothelium that were contracted with 5-HT (1 μM). A maximum effect was consistently observed at the highest concentration studied (100 μM). The results showed differences between the control group (ethanol) and the compounds under study, for the following concentrations: BPA at 0.2 and 2 µM (*p* < 0.01), and at 20 and 100 µM (*p* < 0.001); BPF at 0.02 to 100 µM (*p* < 0.001); BPS at 0.02 µM (*p* < 0.05), 0.2 µM (*p* < 0.01), and 2 to 100 µM (*p* < 0.001). Compared to BPA, BPF exhibited significantly greater vasorelaxant effects at 0.02, 2, 20, and 100 µM. Similarly, BPS demonstrated stronger vasorelaxation, though only at higher concentrations (2, 20 and 100 µM, Table S1).

Figure 1B shows the direct effects of BPA, BPF and BPS (0.002–100 µM) on HUA rings without endothelium, contracted with KCl (60 mM). Again, a maximum effect was observed at the highest concentration under study (100 µM). Statistically significant differences were found between the control group and the study groups for the concentrations: BPA at 0.2 µM (*p* < 0.05), 2 µM (*p* < 0.01), 20 and 100 µM (*p* < 0.001); BPF at 0.02 to 0.2 µM (*p* < 0.05) and 2 to 100 µM (*p* < 0.001); BPS at 0.02 to 100 µM (*p* < 0.001). Compared to BPA, BPF exhibited a higher vasorelaxant effect only at a 100 µM concentration (*p* < 0.001). Meanwhile, BPS showed significantly greater vasorelaxation across all concentrations, with the exception of 2 µM (Table S2).

### 3.2. Effects of Long-Term Exposure to BPA, BPF and BPS on the Contractility of HUA

**Figure 2:**
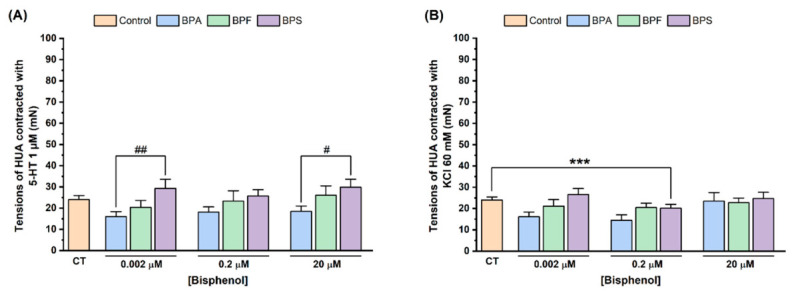
Tension expressed in millinewtons (mN) of arteries incubated with BPA, BPF and BPS (0.002, 0.2 and 20 µM) and contracted with serotonin (5-HT; 1 µM) (**A**) and potassium chloride (KCl; 60 mM) (**B**). The bars represent the mean value and the vertical lines the standard error (S.E.) of at least 4 independent experiments of different HUA (n = 4), with two to three replicates for each bisphenol’s concentration. Statistical analysis was carried out using the one-way ANOVA test, followed by Dunn’s post hoc test, where *** (*p* < 0.001) represents a statistically significant difference compared to the control and # (*p* < 0.05) and ## (*p* < 0.01) represent statistically significant differences when compared to BPA.

To investigate the genomic effects of exposure to different concentrations of BPA, BPF and BPS, artery rings were incubated for 24 h and contracted with 5-HT (1 µM) or KCl (60 mM), and the resulting tensions are presented in Figure 2. The results indicated no differences were observed between BPA and BPF for either contractile agent. However, in the contraction induced by 5-HT (Figure 2A), there are significant differences between BPA and BPS at concentrations of 0.002 µM (*p* < 0.01) and 20 µM (*p* < 0.05). Regarding the KCl-induced contraction (Figure 2B), a statistically significant effect was found between the control group and BPS at 0.2 µM (*p* < 0.001).

### 3.3. Effects of BPA, BPF and BPS on the cGMP Signaling Pathway of HUA

**Figure 3:**
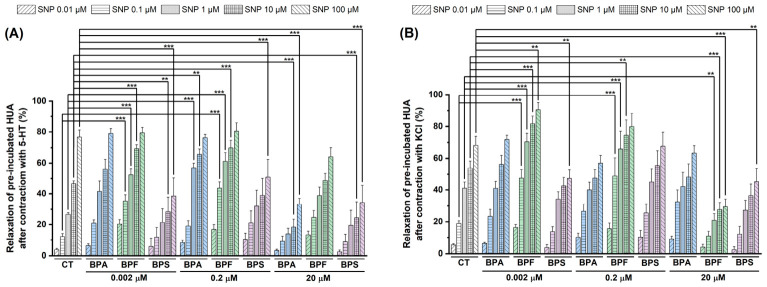
Percentage (%) of relaxation of human umbilical arteries incubated with BPA, BPF and BPS (0.002, 0.2 and 20 µM) and contracted with serotonin (5-HT; 1 µM) (**A**) and potassium chloride (KCl; 60 mM) (**B**), subjected to cumulative concentrations of sodium nitroprusside (SNP; 0.01, 0.1, 1, 10 and 100 µM). The bars represent the mean value and the vertical lines the standard error (S.E.) of at least 4 independent experiments of different HUA (n = 4), with two to three replicates for each bisphenol’s concentration. Statistical analysis was carried out using the two-way ANOVA test, followed by the Holm–Sidak post hoc test, where ** (*p* < 0.01) and *** (*p* < 0.001) represent statistically significant differences when compared to the control.

After incubating HUA rings for 24 h with different concentrations of BPA, BPF and BPS, the involvement of these compounds in the cGMP signaling pathway was analyzed. To this end, the rings were contracted with 5 HT (1 µM) (Figure 3A) and with KCl (60 mM) (Figure 3B) and subjected to different concentrations of sodium nitroprusside (SNP; 0.01, 0.1, 1, 10 and 100 µM), a stimulator of soluble guanylyl cyclase (sGC).

Figure 3A shows statistically significant interactions between the bisphenol incubations and the SNP concentrations analyzed. In the control group (CT) it was found that SNP induced vasorelaxation of the artery, with the maximum effect observed at a concentration of 1 µM SNP. When compared to the control group, the rings incubated with 0.2 µM BPA showed greater relaxation for the 1 and 10 µM SNP concentrations (*p* < 0.001 and *p* < 0.01, respectively). However, when incubated with 20 µM BPA, there was a significant decrease in relaxation for the 10 and 100 µM concentrations of SNP (*p* < 0.001). Arteries incubated with 0.002 and 0.2 µM BPA showed significantly greater relaxation for the 0.1, 1 and 10 µM concentrations of SNP (*p* < 0.001). Concerning BPS, a significant decrease was observed at all incubation concentrations when using 100 µM SNP (*p* < 0.001). There was also a decrease in BPS of 0.002 µM and 20 µM after using 10 µM SNP (*p* < 0.01 and *p* < 0.001, respectively). When comparing BPA and its substitutes, at an incubation concentration of 0.002 µM, BPS shows a decreased vasorelaxant response. When compared with BPA, this decrease was observed for the 1 µM, 10 and 100 µM concentrations of SNP. At the 0.2 µM incubation concentration, the substitutes behave in the same way, with a decrease in the BPS response with 1 and 100 µM of SNP. Finally, at the 20 µM bisphenol concentration, there was an increase in the vasorelaxant response of BPS, with statistically significant differences for the 1 µM, 10 and 100 µM SNP concentrations (Table S3).

In Figure 3B, we can see that in the control group, SNP induced relaxation of the umbilical artery. It can also be seen that there is a gradual decrease in the BPF response, with the greatest statistical differences being observed when comparing the control with BPF at all its incubation concentrations. The HUA incubated with BPF 0.002 µM shows an increased vasorelaxant response at SNP concentrations of 0.1, 1 and 10 µM (*p* < 0.001) and 100 µM (*p* < 0.01). At the 0.2 µM concentration, this increase can still be observed, but only at the 0.1 and 1 µM (*p* < 0.001) and 100 µM (*p* < 0.01) concentrations. However, at the 20 µM BPF concentration, there is a decrease in the vasorelaxant response, with a statistically significant difference at the 1 µM (*p* < 0.01), 10 and 100 µM (*p* < 0.001) concentrations. When comparing the control with the BPS incubations, there was only a statistically significant difference for the highest incubation (20 µM) and the lowest (0.002 µM) at the 100 µM SNP concentration (*p* < 0.01). When comparing the various bisphenols, statistically significant differences were observed only between BPA and BPF across all incubation concentrations. Thus, for BPF 0.002 µM, an increase is observed for SNP concentrations of 0.1, 1, 10 and 100 µM. In the 0.2 µM incubation, an increase in the vasorelaxant response continues to be observed for the 0.1, 1, 10 and 100 µM SNP concentrations. However, for BPF 20 µM, a significant decrease in relaxation was observed for the SNP concentration of 100 µM (Table S4).

### 3.4. Effects of BPA, BPF and BPS on the Activity of L-Type Ca^2+^ Channels of HUA

**Figure 4:**
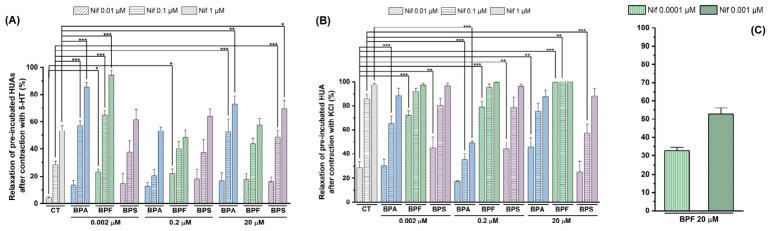
Percentage (%) relaxation of human umbilical arteries incubated with BPA, BPF and BPS (0.002, 0.2 and 20 µM) and contracted with serotonin (5-HT; 1 µM) (**A**) and potassium chloride (KCl; 60 mM) (**B**), subjected to cumulative concentrations of nifedipine (Nif; 0.01, 0.1 and 1 µM). The percentage (%) of relaxation of HUA incubated with BPF 20 µM, contracted with KCl (60 mM) and exposed to cumulative concentrations of Nif (0.00001 and 0.001 µM) is shown in (**C**), with an additive effect observed. The bars represent the mean, and the vertical lines the standard error (S.E.) of at least 4 independent experiments of different HUA (n = 4), with two to three replicates for each bisphenol’s concentration. Statistical analysis was carried out using a two-way ANOVA test, followed by the Holm–Sidak post hoc test, where * (*p* < 0.05), ** (*p* < 0.01) and *** (*p* < 0.001) represent statistically significant differences when compared to the control.

After incubating HUA rings for 24 h with different concentrations of BPA, BPF and BPS, the involvement of Ca2+ channels in the vascular response induced by exposure to these compounds was analyzed. To this end, the rings were contracted with 5-HT (1 µM) (Figure 4A) and with KCl (60 mM) (Figure 4B) and subjected to different concentrations of nifedipine (Nif; 0.01, 0.1 and 1 µM).

As shown in Figure 4A, in the control group, Nif induced vasorelaxation after 5-HT-induced contraction, with the maximum effect observed at 1 µM Nif. When compared to the control group, it was found that the rings incubated with 0.002 µM BPA showed greater relaxation for the 0.1 and 1 µM concentrations of Nif (*p* < 0.001). The rings incubated with 20 µM BPA showed significantly greater relaxation for the same concentrations of the vasorelaxant agent, 0.1 µM (*p* < 0.001) and 1 µM (*p* < 0.01). On the other hand, the rings incubated with BPF 0.002 µM showed significantly greater relaxation for all the concentrations of Nif studied: 0.01 µM (*p* < 0.05), 0.1 and 1 µM (*p* < 0.001). A statistical difference was also observed in the 0.2 µM BPS incubation for the 0.1 µM Nif concentration (*p* < 0.05). The rings incubated with BPS 20 µM showed greater relaxation for the 0.1 and 1 µM concentrations of Nif (*p* < 0.001 and *p* < 0.05, respectively). Regarding the effects of 0.1 µM Nif among the bisphenols, it was observed that relaxation in arteries incubated with 0.2 µM BPA was lower than that of BPF (*p* < 0.001). In contrast, when comparing the 0.1 µM and 1 µM concentrations of Nif in the incubation with 0.002 µM BPA, there was a decrease in relaxation in the arteries incubated with 0.002 µM BPS (*p* < 0.01 and *p* < 0.001, respectively) (Table S5).

As can be seen in Figure 4B, when compared to the control group, the rings incubated with 0.002 µM BPA showed less relaxation for 0.1 µM Nif (*p* < 0.001). The rings incubated with 0.2 µM BPA showed significantly less relaxation for the 0.1 and 1 µM concentrations (*p* < 0.001). When incubated with 20 µM BPA, the 0.01 µM concentration of Nif showed significantly greater relaxation (*p* < 0.01). On the other hand, rings incubated with 20 µM BPF showed a relaxation percentage of approximately 100% across all Nif concentrations. Due to this exacerbated response of Nif with BPF, it was necessary to assess the effects that lower concentrations of Nif would have on the vascular response (Figure 4C). A statistically significant increase was also observed in the 0.002 µM and 0.2 µM BPS incubations for the 0.01 µM Nif concentration (*p* < 0.01). The rings incubated with BPS 20 µM showed significantly less relaxation for the Nif 0.1 µM concentrations (*p* < 0.001) (Table S6).

### 3.5. Direct Vascular Effects of BPA, BPF and BPS on 5-HT-Induced Contractility of HUASMC

**Figure 5:**
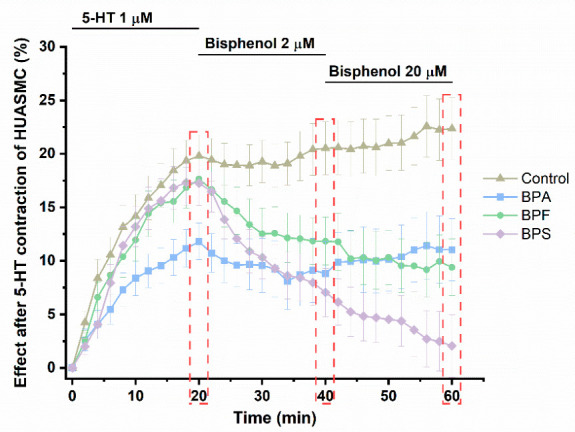
Temporal profile of serotonin-induced contraction, followed by relaxation induced by 2 and 20 µM of BPA, BPF and BPS, in human umbilical artery smooth muscle cells. The results are expressed as a percentage reduction in the compensated area, which evaluates the variation in the area of the cells in relation to the initial area. The dots represent the mean and the vertical lines the standard error (S.E.) of individual SMC (minimum of 12 replicates) from at least 5 different HUAs (n).

The PCSA technique was used to evaluate HUASMC cell contractility. To assess the direct effects of BPA, BPF and BPS (2 and 20 µM) at the cellular level, HUASMC were contracted with 5-HT, 1 µM (Figure 5). The results showed no statistically significant interactions between the compounds and the added 5-HT concentration (*p* = 0.079). However, statistical differences were observed between the control group (ethanol) and the different concentrations of bisphenols, for the concentrations: BPA, BPF and BPS at 2 µM (*p* = 0.005, *p* = 0.019 and *p* < 0.001, respectively) and BPA, BPF and BPS at 20 µM (*p* = 0.012, *p* = 0.007 and *p* < 0.001, respectively) (Table S7). As shown in Figure 5, both BPA and its substitutes, at both concentrations, induced a relaxing effect on HUASMC. These results are in line with those obtained in the study of direct vascular effects on the 5-HT-induced contractility of HUA using the organ bath technique. Furthermore, the relaxing effect on HUASMC induced by BPA (2 and 20 µM) was similar to that induced by BPF and BPS for the same concentrations, with no statistically significant differences between compounds (Table S7).

### 3.6. Genomic Effects of Exposure to BPA, BPF and BPS

**Figure 6:**
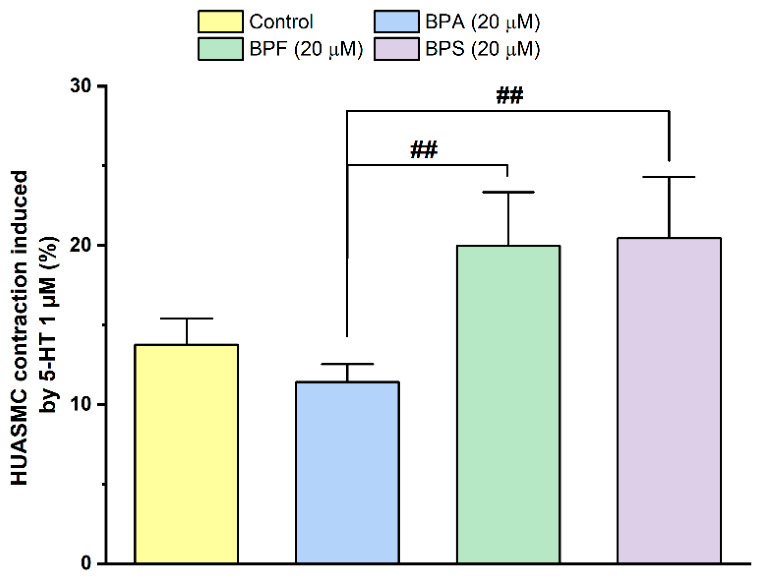
Effect of serotonin (5-HT; 1 µM) on human umbilical artery smooth muscle cells incubated with 2 and 20 µM of BPA, BPF and BPS. The bars represent the mean and the vertical lines the standard error (S.E.) of individual SMC (minimum of 20 replicates) from at least 5 different HUAs (n). Statistical analysis was carried out using the one-way ANOVA test, followed by Dunn’s post hoc test, where ## (*p* < 0.01) represents a statistically significant difference when compared to BPA.

**Figure 7:**
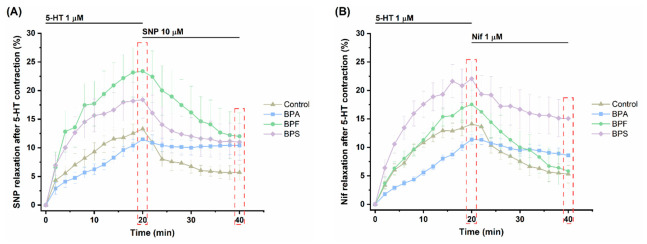
Time profile of contraction induced by serotonin (5-HT; 1 µM), after 24 h exposure to 20 µM of BPA, BPF and BPS, followed by relaxation induced by sodium nitroprusside (SNP; 10 µM) (**A**) and nifedipine (Nif; 1 µM) (**B**), in human umbilical artery smooth muscle cells. The results are expressed as a percentage reduction in the compensated area, which evaluates the variation in the area of the cells in relation to the initial area. The dots represent the mean and the vertical lines the standard error (S.E.) of individual SMC (minimum of 9 replicates) from at least 3 different HUAs (n).

To assess the genomic effects of BPA, BPF and BPS, HUASMC were incubated for 24 h with these bisphenols and subsequently contracted with 5-HT (1 μM) (Figure 6 and Figure 7). For both HUASMC incubated with BPA, BPF and BPS, no statistically significant differences were observed compared to the control group (Figure 6). Thus, it was found that neither compound modified the contractile response to 5-HT. However, compared with BPA, the results showed statistically significant effects for BPF (*p* = 0.004) and BPS (*p* = 0.003) incubations (Figure 6).

Next, the involvement of cyclic nucleotides in the cellular response induced by these compounds was evaluated. To this end, incubated HUASMC were contracted with 5-HT (1 µM) and exposed to the action of SNP (10 µM) (Figure 7A). There were no statistically significant differences between the control and the different bisphenol incubations after the action of sodium nitroprusside (Table S8A).

We also assessed the involvement of Ca^2+^ channels in the cellular response induced by bisphenols after incubating HUASMC with BPA, BPF or BPS (20 μM) for 24 h. To this end, the incubated HUASMCs were contracted with 5-HT (1 µM) and exposed to the action of Nif (1 µM) (Figure 7B). However, after the action of nifedipine, there was a significant increase in the value of the reduction in the compensated area of HUASMC incubated with BPS compared to the control (*p* = 0.013) (Table S8B). Thus, in these cells, the relaxing effect induced by Nif was less than that observed in the control group. Regarding HUASMC incubated with BPA and BPF, the relaxing response induced by Nif was similar to that of the control group, and no significant differences were observed (*p* = 0.432 and *p* = 0.868, respectively) (Table S8B). These results are consistent with those obtained in a study of the involvement of Ca^2+^ channels in bisphenol-induced vasorelaxation using the organ bath technique.

### 3.7. Cell Viability Studies (MTT)

**Figure 8:**
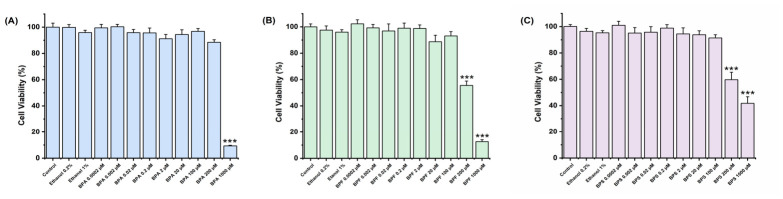
Percentage (%) of HUASMC cell viability under the effect of BPA (**A**), BPF (**B**) and BPS (**C**). Each bar represents the mean value, and the vertical line indicates the standard error (S.E.) of 5 independent experiments, each performed with 4 replicates using HUAs from five different donors. Statistical analysis was performed using the one-way ANOVA test, followed by Dunn’s post hoc test, where *** (*p* < 0.001) represents statistically significant differences when compared to the control.

Figure 8 illustrates the effects of BPA, BPF and BPS (0.002–1000 µM) on the viability of HUASMC. Regarding BPA (Figure 8A), only the highest concentration (1000 µM) caused a significant decrease in cell viability (*p* < 0.001). However, for its substitutes BPF and BPS (Figure 8B and Figure 8C, respectively), this decrease was observed for the two highest concentrations (200 µM and 1000 µM). Specifically, the highest concentration of BPF resulted in only 12.57% viability, while the highest concentration of BPS showed 41.81% viability. For all other concentrations of BPA (0.0002, 0.002, 0.02, 0.2, 2, 20, 100, 200 µM) and its substitutes (0.0002, 0.002, 0.02, 0.2, 2, 20 and 100 µM), no significant differences were found compared to the control and the vehicle groups, indicating that these concentrations do not induce cell toxicity (Table S9).

### 3.8. Gene Expression

**Figure 9:**
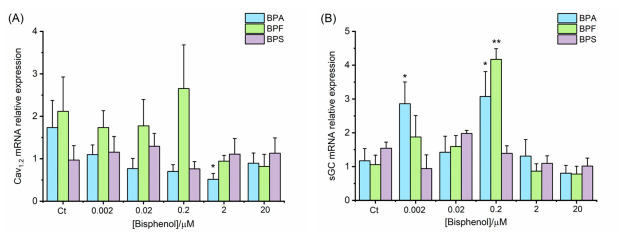
Levels of *mRNA* relative expression of (**A**) *Cav1.2* channels and (**B**) *sGC* in HUASMC after 24 h exposure to BPA, BPF and BPS (0.002–20 µM). *Human β-actin* was used as the housekeeping gene to normalize the mRNA expression of *Ca_V_1.2* and *sGC*. Each bar represents the mean value, and the vertical line indicates the standard error (S.E.) of at least 3 different HUAs with 2 replicates. * Represents statistical differences in comparison to control (Ct: solvent control) using a one-way ANOVA test, followed by Dunn’s post hoc test or Holm–Sidak test, where * (*p* < 0.05) and ** (*p* < 0.01).

The relative mRNA expression of calcium channels (*Ca_V_1.2*) and soluble guanylate cyclase (*sCG*) after HUASMC 24 h-exposure to BPA, BPF and BPS (0.002–20 µM) is presented in Figure 9. Regarding the *Ca_V_1.2* channels, there was only a significant decrease in the mRNA expression of HUASMC incubated with 2 μM of BPA (*p* = 0.031) compared to the control (Figure 9A). For the *sGC* expression, illustrated in Figure 9B, there was a significant increase in HUASMC incubated with 0.002 μM of BPA (*p* = 0.021), 0.2 μM of BPA (*p* = 0.010) and 0.2 μM of BPF (*p* = 0.009) when compared with the control.

## 4. Discussion

Over the years, BPA substitutes—such as BPF and BPS—have been replacing BPA in several everyday products, since exposure to this compound has been associated with the development of several pathologies, namely in the cardiovascular system [51]. However, questions have recently been raised about the safety and benefits of this substitution. Indeed, some studies have already associated exposure to these BPA substitutes with disruption of the normal function and morphology of the cardiovascular system. For example, exposure to bisphenols has been associated with changes in heart rate [52,53], size of various regions of the heart [54,55,56], and alterations in Na^+^, Ca^2+^ and K^+^ currents [41,52].

Therefore, the main objective of this study was to evaluate the effects of BPF and BPS on the vasculature of pregnant women and to determine whether they are more harmful than those caused by BPA. For this purpose, in the first phase, arterial contractility studies were performed in HUA using the organ bath technique. Therefore, the endothelium was previously removed from the HUA to ensure that the observed effects were solely due to BPA, BPF, or BPS acting on the smooth muscle.

Initially, studies were conducted to evaluate the direct vascular effects of these bisphenols on HUA contractility. For this purpose, increasing and cumulative concentrations of BPA, BPF, BPS or ethanol (control) were added to non-incubated HUA rings contracted with 5-HT or KCl. In both cases, the results showed that the effects induced by the added compounds depend on the concentration since the interaction between the two factors (BPA/BPS/BPF/ethanol vs. added concentration) was observed. Overall, all three bisphenols induced short-term and concentration-dependent relaxations in endothelium-free HUA when these were contracted by both vasoactive agents. Furthermore, the results have shown that these bisphenols can cause rapid and reversible effects. Since endothelium was not present in these HUA rings, it can be concluded that the vasorelaxant effects observed are not related to the endothelial synthesis of nitric oxide (NO), i.e., these effects are endothelium-independent. These results are in agreement with what was observed in a study performed by our research group in which the rapid effects of BPA on HUA without endothelium were also evaluated [10]. The use of endothelium-denuded arteries, whilst necessary to focus on the intrinsic mechanisms of BPA and its substitutes in the smooth muscle, excludes the physiological contribution of endothelium-derived NO and other endothelial factors. Consequently, the results reflect the behavior of smooth muscle in the absence of this essential modulatory layer, a crucial step before investigating the integrated vascular responses in intact vessels, where there is a continuous and dynamic interaction between the endothelium and smooth muscle cells. Nevertheless, in future studies should be important to analyze the endothelium effect in the bisphenols’ action in the HUA to better simulate the in vivo environment. Performing experiments with HUA rings with intact endothelium would be a good strategy in determining whether BPs inhibit endothelium-dependent relaxation, including the application of specific inhibitors of endothelial pathways.

On the other hand, it was verified that the maximum relaxation induced by bisphenols was achieved for the maximum added concentration (100 μM). Comparing the vasorelaxant effects between BPA and its substitutes, both BPF and BPS showed superior vasorelaxant effects for all concentrations tested when HUA was contracted with 5-HT and with KCl. Furthermore, when the effects caused by the two BPA substitutes were compared, it was found that for both contractile agents, the concentration of BPF 100 μM induced a higher relaxation in HUA rings than BPS 100 μM.

Indeed, these results are consistent with the study conducted by Tuzimski, Szubartowski, Stupak, Kwasniewski, Szultka-Mlynska, Kwasniewska and Buszewski [34] on rat aortic arteries contracted by noradrenaline, in which 14 different bisphenols were analyzed. These authors observed that, also in rat aortic arteries contracted with noradrenaline, the most potent vasodilator activities, with EC_50_ values of 57.2 μM and 61.9 μM, were observed for bisphenols AF and AP. On the other hand, the authors also demonstrated that acute and chronic in vivo exposure in rats was not consistent, since the impact of bisphenol AF on blood pressure was mild, and the measurement of the most sensitive marker of cardiotoxicity, cardiac troponin T, also did not indicate cardiotoxicity [34].

Concordantly, cellular-level results using the PCSA technique showed that the effect induced by bisphenols is concentration-dependent. Moreover, also in HUASMC, BPA, BPF and BPS induced significant relaxant effects, similar to each other. Therefore, these results suggest that both BPA and its substitutes can induce a direct relaxation in HUASMC. These results are in agreement with different studies performed with other EDCs at the cellular level [48,57].

On the other hand, recent studies have documented the accumulation of BPA substitutes in maternal plasma, umbilical cord and placenta samples [33,36,58,59,60], suggesting their bioaccumulation in these tissues and a possible maternal-fetal transfer, as already evidenced in animal models [61,62,63] and humans [33,36,58]. Thus, it can be hypothesized that, in addition to the rapid vasorelaxant effects induced by bisphenols, these compounds could also exert long-term effects on the pregnant woman and their fetus as a result of this bioaccumulation.

Based on this hypothesis, the next step of this work was to assess the long-term, i.e., the genomic effects associated with bisphenol exposure. For this purpose, some endothelium-free HUA rings were incubated with BPA, BPF or BPS (0.002, 0.2 and 20 μM) for 24 h and then were contracted with the vasoactive agents 5-HT and KCl. Regarding 5-HT-induced contractions, HUA rings incubated with BPA and BPS show different vascular responses, which may be related to their effect on 5-HT receptors (5-HT_2A_, 5-HT_1B_/5-HT_1D_ and 5-HT_7_). Effects on 5-HT receptors were also implicated in endocrine-disrupting effects at the vascular level promoted by other EDCs, namely tributyltin (TBT; 100 μM) [40] and tetrabromobisphenol A (2,6-dibromo-4-[2-(3,5-dibromo-4-hydroxyphenyl)propan-2-yl]phenol; TBBPA; 50 μM) [64].

Moreover, regarding KCl-induced contractions, it was observed that when HUA rings were incubated with BPA 0.2 μM, their contractile capacity also decreased compared to the control. Therefore, these results suggest that the contractile capacity of HUA depends on exposure to different concentrations of this bisphenol and could modulate vascular homeostasis by interfering with Ca^2+^ channels, namely LTCC. In other words, these BPA concentrations might decrease the expression or opening of these channels, thereby reducing Ca^2+^ influx and, consequently, the contractile capacity of HUA [46]. It should also be noted that similar results have already been observed for HUA incubated for 24 h with TBBPA 50 μM [64]. Regarding BPF and BPS, none of the incubations altered the vasocontractile response induced by both vasoactive agents compared to the control. These first results seem to suggest that BPF and BPS may be less harmful to the cardiovascular system than BPA, as suggested by Prudencio, Swift, Guerrelli, Cooper, Reilly, Ciccarelli, Sheng, Jaimes and Posnack [52].

In a second phase, the involvement of cyclic nucleotides and Ca^2+^ channels in the vasorelaxation of HUA induced by BPA, BPF and BPS was evaluated, since the main mechanisms responsible for this process occur by activation of the cGMP signaling pathway and/or by activation/inhibition of ion channels [39]. To evaluate the involvement of cyclic nucleotides, the effects of different concentrations of SNP (a stimulator of sGC) were studied in HUA incubated with BPA, BPF or BPS (0.002, 0.2 and 20 μM) and contracted with 5-HT or KCl. In non-incubated arteries contracted with 5-HT or KCl, SNP induced a relaxant effect as observed in other studies [10,39,64,65].

Regarding arteries incubated with BPA, BPF or BPS and contracted with 5-HT, the results showed that the SNP-induced effect depends on the concentration of bisphenols incubated, as an interaction between the two factors (incubation with BPA/BPF/BPS vs. SNP concentration) was observed. Overall, in the SNP results, we can observe that the BP effects depend on the concentration of SNP used, the type of BP incubated, and the contractile agent used. Furthermore, we can conclude that, in 5-HT-induced contractions, BPS appears to inhibit the cGMP signaling pathway (NO/sGC/cGMP/PKG) more prominently than the other BPs and the control. With regard to KCl-induced concentrations, BPF appears to have a dual effect: the relaxing effect is more pronounced at lower incubation concentrations and diminishes at higher concentrations. In the case of BPS, this pattern is not observed; instead, an inverted U-shaped response (even weak) is evident. These results are in accordance with gene expression data, in which the effect of BPS was similar to the control, and BPF exhibits a dual response in HUASMC (an up-regulation trend in the lowest concentrations and a down-regulation trend in the highest concentrations). However, a significant effect was only observed for 0.2 µM BPF exposure. Moreover, a higher mRNA gene expression was also observed for 0.002 and 0.2 µM BPA exposure, as obtained by Fonseca et al. [10].

In 2022, Fatai et al. also discovered in 80 male Wistar rats that after oral exposure to BPF (10, 30 and 50 mg/kg), plasma concentrations of NO and cGMP in the smooth muscle of the corpus cavernosum of the penis were decreased in all exposed groups [66]. Furthermore, they verified that these changes were directly dose-dependent (at higher doses, the reduction was greater) and did not appear to be reversible after 28 days of treatment cessation. Thus, the authors suggested that BPF, by disrupting this signaling pathway, may negatively affect the quality of sexual performance and consequently the quality of the offspring [66]. On the other hand, a decrease in NO production after exposure to BPA (10 and 25 mg/kg) has also been observed in Wistar male rat hearts [67]. This alteration may promote an increase in oxidative stress and subsequently the development of cardiovascular diseases (CVD), namely, hypertension [67].

On the other hand, to evaluate the involvement of Ca^2+^ channels, namely LTCC, in bisphenol-induced vasorelaxation of HUA, the effects of different concentrations of Nif (LTCC-specific antagonist) were studied in HUA incubated with BPA, BPF or BPS (0.002, 0.2 and 20 μM) and contracted with 5-HT and KCl. In non-incubated arteries contracted with 5-HT or KCl, Nif was found to induce a relaxant effect, as has been observed in other studies [39,64]. Regarding arteries incubated with BPA, BPF or BPS and contracted with 5-HT, the results showed that the effect induced by Nif depends on the concentration of bisphenols incubated, as an interaction between the two factors (incubation with BPA/BPF/BPS vs. Nif concentration) was observed. In 5-HT-induced contractions, we observed an increase in Nif-induced vasorelaxation at the lowest incubation concentrations (0.002 µM) of BPA and BPF. Conversely, the maximum incubation concentration of BPS (20 µM) increased Nif-induced vasorelaxation, suggesting possible different modes of action between the two alternative BPs. Moreover, in HUA incubated with BPA, a non-linear relationship was found between the incubation dose and the observed effects, i.e., a typical non-monotonic response was observed [41,68,69]. These results are in line with the study performed by Feiteiro et al. where it was verified that in SMCs of the A7r5 cell line, BPA inhibited the Ca^2+^ currents associated with LTCC in a non-linear manner [41]. This complicates the definition of exposure dose limits that do not lead to more harmful effects on human health, since it has been observed that exposure to low concentrations of this bisphenol may be associated with changes in the vasorelaxation of HUA. Indeed, this may be of particular concern, particularly in the most vulnerable stages such as pregnancy [4]. On the other hand, in the case of KCl-induced contractions, there appears to be a potentiation of the relaxing effect of Nif for all BPs, with this effect being most evident with BPF (20 µM). Additionally, gene expression studies were also concordant with organ bath data, demonstrating a higher (not significant) mRNA expression of Ca^2+^ channels in the 0.2 μM BPF exposure. Contrarily, a reduced effect of 2 μM BPA on Ca^2+^ channels mRNA expression was attained, which is in accordance with Fonseca et al. [10].

Indeed, these bisphenols seem to exhibit the same mode of action concerning the Nif response and thereby modulate the vasorelaxation. Over the years, some studies have evaluated the effects that exposure to BPA may have on Ca^2+^ homeostasis [41,70,71,72]. Overall, through patch-clamp experiments, it was verified that BPA was able to inhibit ion channels present in several types of cells, such as SMCs of the A7r5 cell line and excised rat hearts, inhibiting Ca^2+^ currents [41,70,71,72]. Similar results were obtained by Prudencio et al. for BPA, BPF and BPS [52]. These authors found that in these cells both BPA and BPF inhibited LTCC activity, thus inhibiting its ionic currents [52]. Thus, according to these investigators, the results of this study may also suggest that both BPA and BPF, by disturbing Ca^2+^ influx, may impair the vascular homeostasis of HUA and, consequently, promote the development of CVD, since changes in the homeostasis of this ion have already been associated with the development of these pathologies [39].

Finally, the involvement of cyclic nucleotides and Ca^2+^ channels in the relaxation of HUASMC was also evaluated. More specifically, the effects induced by SNP 10 μM and Nif 1 μM were evaluated in HUASMC incubated for 24 h with BPA, BPF or BPS 20 μM. It was found that the results obtained were in line with those observed by the organ bath technique in this study and others [10], which reinforces the robustness of the data. As observed in HUASMC incubated with BPA 20 μM, the cells incubated with BPF and BPS 20 μM did not exhibit an SNP-induced relaxation significantly different from the control. These data seem to suggest that in this situation, the effects caused by both BPA substitutes are similar. In contrast, Nif-induced relaxation of HUASMC incubated with BPS 20 μM was lower than that observed for HUASMC incubated with BPA and BPF 20 μM. Therefore, these data seem to suggest that BPS may disrupt mechanisms associated with Ca^2+^ influx. In the study performed by Gao et al., exposure to low concentrations of this EDC was associated with the disruption of Ca^2+^ homeostasis in ventricular myocytes isolated from Sprague-Dawley rat hearts [53]. However, in another study, exposure to low concentrations of this BPA substitute was associated with decreased contraction and relaxation rates of myocytes isolated from CD-1 mice hearts [73]. On the other hand, in the work performed by Prudencio, Swift, Guerrelli, Cooper, Reilly, Ciccarelli, Sheng, Jaimes and Posnack [52], it was verified that BPS did not alter any of the cardiac electrophysiology parameters tested. Thus, it is clear that the studies performed so far are not very concordant regarding the potential effects of BPS on vascular homeostasis, so further research is needed. In addition, the differences in direct vascular effects and genomic effects highlight the distinct mechanisms underlying acute functional activity and long-term genomic effects, since a compound may be a potent acute modulator of vascular tone whilst exhibiting a less adverse profile regarding genomic disruption. Therefore, the safety assessment of BPA substitutes must consider both acute functional outcomes and genomic effects separately [10,40].

Concerning the cell viability results, only the highest concentration of BPA (1000 µM) resulted in a significant decrease in cell viability of approximately 91%. However, for its substitutes, a decrease in cell viability was observed at the two highest concentrations (200 and 1000 µM). Compared to the control, BPF showed a decrease of approximately 44% (200 µM) and 87% (1000 µM), while BPS showed a decrease of approximately 40 per cent (200 µM) and 58 per cent (1000 µM). Aiming to investigate whether BPF and BPS compounds are suitable alternatives to BPA, Shao et al. performed acute cytotoxicity tests in U2OS cells exposed to BPA, BPF and BPS in increasing concentrations (0.39 μM to 100 μM) for 24 h. The results showed that exposure to the three substances caused a significant and concentration-dependent reduction in cell viability. At a concentration of 100 μM, it was observed that BPA induced a survival rate of approximately 8%, while BPF induced a survival rate of approximately 31% and BPS of 58% [74]. Another relevant study was carried out by Martínez et al. to compare the toxic effects on cell viability and obesogenic activity of bisphenol A (BPA) and its analogs, bisphenol S (BPS) and bisphenol F (BPF). Undifferentiated 3T3-L1 preadipocyte cells were exposed to bisphenols at concentrations ranging from 0 to 400 μM over 24 h. The results indicated that, after exposure, only treatment with BPA at a concentration of 400 μM resulted in a significant reduction in cell viability, of approximately 16 per cent, compared to the control. In contrast, neither BPS nor BPF showed detectable cytotoxic effects under the same experimental conditions, even at the highest concentration tested (400 μM) [75]. Although the results are consistent with those of the studies mentioned regarding the reduction in cell viability at high concentrations, it should be noted that the functional effects on vascular contractility occurred at substantially lower, non-cytotoxic concentrations. These results may be due to the type of exposure, since in vivo, chronic exposure to lower concentrations may have different effects from acute exposure to very high concentrations in vitro. Furthermore, this indicates that the altered vascular reactivity is a specific signaling-mediated effect, distinct from general cytotoxicity, and suggests a wide safety margin for acute exposure in terms of cell survival.

In summary, this study demonstrated that BPA, BPF and BPS induce non-genomic effects on HUA rings. Indeed, the results of this study suggest that these bisphenols can induce short-term, concentration-dependent relaxations in HUA without endothelium, and this effect was greater for BPA substitutes. At the genomic level, the results indicate that the contractile mechanism of HUA incubated with BPA was altered, suggesting that BPF and BPS may be less harmful to the cardiovascular system than BPA. However, the data from this study also suggest that at the genomic level, both BPA and BPF appear to alter the main mechanisms responsible for the relaxation of HUA. On several occasions, the vasorelaxant response was found to be more modified after exposure to BPF than to BPA. Finally, preliminary data from PCSA experiments suggest that BPS may also have some genomic effects on HUASMC, namely regarding the involvement of Ca^2+^ channels, which might be associated with the development of cardiovascular pathologies. Thus, these results demonstrate that these substitutes do not appear to be safer for cardiovascular health, which is one of the purposes for which they were developed. Thus, the synthesis of new, safer substitutes for human health, in particular cardiovascular health, is proposed. Further studies should continue to be developed to better understand the modes of action of BPA alternatives (BPF and BPS) at the vascular level. Further investigations are suggested, including screening for inhibitors of intracellular signaling pathways to determine if pathways other than cGMP and Ca^2+^ channels are involved, for example, by using various signaling inhibitors such as potassium channel blockers or PKA pathway inhibitors. Furthermore, considering the fundamental role of NO produced by endothelial cells in the vascular regulation exerted by smooth muscle, the potential induction of endothelial disruption by BPs would be a valuable study. Finally, it is important to note that humans are simultaneously exposed to several environmental chemicals daily (rather than a single compound individually), leading to a more complex exposure scenario. Consequently, exposure to BPs ends up being widespread, multi-source, and multi-route, involving the possibility of interactions between them (synergistic, additive, or antagonistic effects), which makes the challenge of studying endocrine disruption even greater [76,77]. In this study, the assessment of the disruptive effects of BPs was explored for the first time at the individual level, but the need to proceed with further investigations targeting mixtures of these chemical stressors is recognized.

## 5. Conclusions

This study demonstrated that exposure to BPA and its substitutes, BPF and BPS, alters the contractile response of HUA at the arterial and cellular levels. At the non-genomic level, the results indicated that all three bisphenols induced a concentration-dependent short-term relaxation, with the effects being more pronounced for the substituents than for BPA. At the genomic level, the results suggest that BPA, BPF, and BPS may interfere with the main pathways responsible for vasorelaxation in HUA, NO/sGC/cGMP/PKG and activation/inhibition of ion channels.

Overall, the results seem to reveal that long-term exposure to BPF may modify the vasorelaxant response of HUA more than BPA and BPS. Therefore, replacing BPA with these analogs does not seem to benefit vascular health, since these data suggest that these bisphenols may induce modulation of the vascular response—though with some differences—particularly during periods of increased susceptibility, such as pregnancy. These changes raise significant concerns, as they may increase maternal and fetal morbidity and mortality rates.

Thus, it is essential to continue evaluating the vascular effects of these EDCs in the future to clarify their modes of action and future implications on maternal-fetal health.

## Data Availability

The original contributions presented in this study are included in the article/Supplementary Materials. Further inquiries can be directed to the corresponding author.

## Supplementary Materials

The following supporting information can be downloaded at https://www.mdpi.com/article/10.3390/jox16030111/s1, Table S1: Statistical differences in the percentage of relaxation of HUA contracted with 5-HT (1 µM), after exposure to cumulative concentrations of BPA, BPF and BPS (0.002–100 µM). The differences considered statistically significant by the two-way ANOVA test, followed by the Holm–Sidak post hoc test, are shown in bold; Table S2. Statistical differences in the percentage of relaxation of HUA contracted with KCl (60 mM) after exposure to cumulative concentrations of BPA, BPF and BPS (0.002–100 µM). The differences considered statistically significant by the two-way ANOVA test, followed by the Holm–Sidak post hoc test, are shown in bold; Table S3. Statistical differences in the percentage of relaxation of HUA incubated with BPA, BPF and BPS (0.002, 0.2 and 20 µM) contracted with 5-HT (1 µM) and subjected to cumulative concentrations of SNP (0.01–100 µM). The differences considered statistically significant by the two-way ANOVA test, followed by the Holm–Sidak post hoc test, are shown in bold; Table S4. Statistical differences in the percentage relaxation of HUA incubated with BPA, BPF and BPS (0.002, 0.2 and 20 µM), contracted with KCl (60 mM) and subjected to cumulative concentrations of SNP (0.01–100 µM). The differences considered statistically significant by the two-way ANOVA test, followed by the Holm–Sidak post hoc test, are shown in bold; Table S5. Statistical differences in the percentage of relaxation of HUA incubated with BPA, BPF and BPS (0.002, 0.2 and 20 µM). contracted with 5-HT (1 µM) and subjected to cumulative concentrations of Nif (0.01–1 µM). The differences considered statistically significant by the two-way ANOVA test, followed by the Holm–Sidak post hoc test, are shown in bold; Table S6. Statistical differences in the percentage of relaxation of HUA incubated with BPA, BPF and BPS (0.002, 0.2 and 20 µM) contracted with KCl (60 mM) and subjected to cumulative concentrations of Nif (0.01–1 µM). The differences considered statistically significant by the two-way ANOVA test, followed by the Holm–Sidak post hoc test, are shown in bold; Table S7. Statistical differences in the time profile of contraction induced by 5-HT (1 µM; 20 min), followed by relaxation induced by BPA, BPF and BPS (2 and 20 µM; 40 and 60 min, respectively) in HUASMC. The differences considered statistically significant by the one-way ANOVA test, followed by the Holm–Sidak post hoc test, are shown in bold; Table S8. Statistical differences in the time profile of contraction induced by 5-HT (1 µM), in HUASMC incubated with BPA, BPF and BPS (20 µM) and subjected to a concentration of SNP (10 µM) (A) and Nif (1 µM) (B). The differences considered statistically significant by the one-way ANOVA test, followed by Dunn’s post hoc test (A) and the Holm–Sidak post hoc test (B) are shown in bold; Table S9. Statistical differences in HUASMC cell viability after the effect of BPA, BPF and BPS (0.0002–1000 µM). Statistically significant differences by the one-way ANOVA test, followed by Dunn’s post-hoc test, are shown in bold.
