# Supplementary material for: Vascular Endocrine-Disrupting Effects of Bisphenol F and Bisphenol S on Human Umbilical Artery
Source: J Xenobiot. 2026 Jun 13;16(3):111. doi: 10.3390/jox16030111 (PMC13300960; doi:10.3390/jox16030111)
Supplement: Supplementary file 1 [file jox-16-00111-s001.zip › jox-4346478-supplementary.pdf]

Supplementary Materials: Vascular Endocrine-Disrupting Effects of Bisphenol F and Bisphenol S on Human Umbilical Artery

Fatima Abrantes-Soares, Mariana Marques Santos, Melissa Mariana, Margarida Lorigo and Elisa Cairrao

Table S1. Statistical differences in the percentage of relaxation of HUA contracted with 5-HT (1 μM), after exposure to cumulative concentrations of BPA, BPF and BPS (0.002 - 100 μM). The differences considered statistically significant by the two-way ANOVA test, followed by the Holm-Sidak post-hoc test, are shown in bold.

	**0.002 μM**	**0.02 μM**	**0.2 μM**	**2 μM**	**20 μM**	**100 μM**
**Control vs BPA**	p=0.924	p=0.179	**p=0.004**	**p=0.002**	**p<0.001**	**p<0.001**
**Control vs BPF**	p=0.112	**p<0.001**	**p<0.001**	**p<0.001**	**p<0.001**	**p<0.001**
**Control vs BPS**	p=0.446	**p=0.020**	**p=0.003**	**p<0.001**	**p<0.001**	**p<0.001**
**BPA vs BPF**	p=0.149	**p=0.020**	p=0.051	**p=0.011**	**p<0.001**	**p<0.001**
**BPA vs BPS**	p=0.516	p=0.321	p=0.469	**p=0.003**	**p<0.001**	**p<0.001**

Table S2. Statistical differences in the percentage of relaxation of HUA contracted with KCl (60 mM), after exposure to cumulative concentrations of BPA, BPF and BPS (0.002 - 100 μM). The differences considered statistically significant by the two-way ANOVA test, followed by the Holm-Sidak post-hoc test, are shown in bold.

	**0.002 μM**	**0.02 μM**	**0.2 μM**	**2 μM**	**20 μM**	**100 μM**
**Control vs BPA**	p=0.522	p=0.152	**p=0.015**	**p=0.002**	**p<0.001**	**p<0.001**
**Control vs BPF**	**p=0.037**	**p=0.026**	**p=0.011**	**p<0.001**	**p<0.001**	**p<0.001**
**Control vs BPS**	**p<0.001**	**p<0.001**	**p<0.001**	**p<0.001**	**p<0.001**	**p<0.001**
**BPA vs BPF**	p=0.183	p=0.281	p=0.600	p=0.225	p=0.445	**p<0.001**
**BPA vs BPS**	**p=0.016**	**p=0.036**	**p=0.046**	p=0.107	**p=0.037**	**p<0.001**

Table S3. Statistical differences in the percentage of relaxation of HUA incubated with BPA, BPF and BPS (0.002; 0.2 and 20 μM). contracted with 5-HT (1 μM) and subjected to cumulative concentrations of SNP (0.01 - 100 μM). The differences considered statistically significant by the two-way ANOVA test, followed by the Holm-Sidak post-hoc test, are shown in bold.

		**0.01 μM**	**0.1 μM**	**1 μM**	**10 μM**	**100 μM**
**0.002 μM**	**Control vs BPA**	p=0.986	p=0.497	p=0.058	p=0.289	p=0.957
	**Control vs BPF**	p=0.052	**p<0.001**	**p<0.001**	**p<0.001**	p=0.957
	**Control vs BPS**	p=0.986	p=0.952	p=0.527	**p=0.005**	**p<0.001**
	**BPA vs BPF**	p=0.649	p=0.457	p=0.663	p=0.339	p=1.000

	**BPA vs BPS**	p=1.000	p=0.892	**p=0.016**	**p<0.001**	**p<0.001**
	**Control vs BPA**	p=0.943	p=0.661	**p<0.001**	**p=0.005**	p=0.957
	**Control vs BPF**	p=0.209	**p<0.001**	**p<0.001**	**p<0.001**	p=0.947
**0.2 μM**	**Control vs BPS**	p=0.782	p=0.457	p=0.527	p=0.289	**p<0.001**
	**BPA vs BPF**	p=0.995	**p=0.003**	p=0.927	**p<0.001**	p=1.000
	**BPA vs BPS**	p=1.000	p=1.000	**p<0.001**	**p<0.001**	**p<0.001**
	**Control vs BPA**	p=0.986	p=0.918	p=0.124	**p<0.001**	**p<0.001**
	**Control vs BPF**	p=0.562	p=0.220	p=0.154	p=0.722	p=0.161
**20 μM**	**Control vs BPS**	p=0.986	p=0.918	p=0.506	**p<0.001**	**p<0.001**
	**BPA vs BPF**	p=0.974	p=0.323	**p=0.002**	**p<0.001**	**p<0.001**
	**BPA vs BPS**	p=1.000	p=1.000	p=0.927	p=0.890	p=1.000

Table S4. Statistical differences in the percentage relaxation of HUA incubated with BPA, BPF and BPS (0.002; 0.2 and 20 μM). contracted with KCl (60 mM) and subjected to cumulative concentrations of SNP (0.01 - 100 μM). The differences considered statistically significant by the two-way ANOVA test, followed by the Holm-Sidak post-hoc test, are shown in bold.

		**0.01 μM**	**0.1 μM**	**1 μM**	**10 μM**	**100 μM**
	**Control vs BPA**	p=0.990	p=0.788	p=0.998	p=0.915	p=0.831
	**Control vs BPF**	p=0.549	**p<0.001**	**p<0.001**	**p<0.001**	**p=0.005**
**0.002 μM**	**Control vs BPS**	p=0.990	p=0.788	p=0.785	p=0.309	**p=0.005**
	**BPA vs BPF**	p=0.997	**p=0.021**	**p=0.001**	**p=0.009**	p=0.142
	**BPA vs BPS**	p=1.000	p=0.925	p=0.994	p=0.564	**p=0.008**
	**Control vs BPA**	p=0.980	p=0.788	p=0.998	p=0.829	p=0.335
	**Control vs BPF**	p=0.473	**p<0.001**	**p<0.001**	**p=0.002**	p=0.173
**0.2 μM**	**Control vs BPS**	p=0.975	p=0.788	p=0.928	p=0.915	p=0.921
	**BPA vs BPF**	p=1.000	**p=0.019**	**p=0.002**	**p=0.001**	**p=0.011**
	**BPA vs BPS**	p=1.000	p=0.999	p=0.998	p=0.975	p=0.720
	**Control vs BPA**	p=0.985	p=0.194	p=0.998	p=0.829	p=0.816
	**Control vs BPF**	p=0.990	p=0.788	**p=0.014**	**p<0.001**	**p<0.001**
**20 μM**	**Control vs BPS**	p=0.990	p=0.788	p=0.138	**p=0.028**	**p=0.002**
	**BPA vs BPF**	p=1.000	p=0.052	p=0.053	p=0.067	**p<0.001**
	**BPA vs BPS**	p=1.000	p=0.052	p=0.391	p=0.715	p=0.115

Table S5. Statistical differences in the percentage of relaxation of HUA incubated with BPA, BPF and BPS (0.002; 0.2 and 20 μM). contracted with 5-HT (1 μM) and subjected to cumulative concentrations of Nif (0.01 - 1 μM). The differences considered statistically significant by the two-way ANOVA test, followed by the Holm-Sidak post-hoc test, are shown in bold.

		0.01 μM	0.1 μM	1 μM
**0.002 μM**	**Control vs BPA**	p=0.141	**p<0.001**	**p<0.001**
	**Control vs BPF**	**p=0.012**	**p<0.001**	**p<0.001**
	**Control vs BPS**	p=0.141	p=0.234	p=0.428
	**BPA vs BPF**	p=0.990	p=0.893	p=0.858
	**BPA vs BPS**	p=1.000	**p=0.020**	**p<0.001**
**0.2 μM**	**Control vs BPA**	p=0.206	p=0.234	p=0.998
	**Control vs BPF**	**p=0.018**	p=0.182	p=0.783
	**Control vs BPS**	p=0.058	p=0.234	p=0.185
	**BPA vs BPF**	p=0.990	**p=0.038**	p=0.983
	**BPA vs BPS**	p=1.000	p=0.052	p=0.557
**20 μM**	**Control vs BPA**	p=0.141	**p<0.001**	**p=0.004**
	**Control vs BPF**	p=0.126	**p=0.049**	p=0.783
	**Control vs BPS**	p=0.141	**p=0.001**	**p=0.018**
	**BPA vs BPF**	p=1.000	p=0.870	p=0.205
	**BPA vs BPS**	p=1.000	p=0.969	p=0.983

Table S6. Statistical differences in the percentage of relaxation of HUA incubated with BPA, BPF and BPS (0.002; 0.2 and 20 μM), contracted with KCl (60 mM) and subjected to cumulative concentrations of Nif (0.01 - 1 μM). The differences considered statistically significant by the two-way ANOVA test, followed by the Holm-Sidak post-hoc test, are shown in bold.

		0.01 μM	0.1 μM	1 μM
**0.002 μM**	**Control vs BPA**	p=0.786	**p<0.001**	p=0.404
	**Control vs BPF**	**p<0.001**	**p=0.393**	p=0.991
	**Control vs BPS**	**p=0.002**	p=0.393	p=0.991
	**BPA vs BPF**	**p<0.001**	**p<0.001**	p=0.920
	**BPA vs BPS**	**p=0.046**	**p=0.045**	p=0.928
**0.2 μM**	**Control vs BPA**	p=0.067	**p<0.001**	**p<0.001**
	**Control vs BPF**	**p<0.001**	p=0.138	p=0.978
	**Control vs BPS**	**p=0.003**	p=0.393	p=0.991
	**BPA vs BPF**	**p<0.001**	**p<0.001**	**p<0.001**

	**BPA vs BPS**	**p<0.001**	**p<0.001**	**p<0.001**
	**Control vs BPA**	**p=0.002**	**p<0.001**	p=0.242
	**Control vs BPF**	**p<0.001**	**p=0.004**	p=0.974
**20 μM**	**Control vs BPS**	p=0.683	**p<0.001**	p=0.277
	**BPA vs BPF**	**p<0.001**	**p<0.001**	p=0.187
	**BPA vs BPS**	**p<0.001**	**p=0.003**	p=1.000

Table S7. Statistical differences in the time profile of contraction induced by 5-HT (1 μM; 20 minutes), followed by relaxation induced by BPA, BPF and BPS (2 and 20 μM; 40 and 60 minutes, respectively) in HUASMC. The differences considered statistically significant by the one-way ANOVA test, followed by the Holm-Sidak post-hoc test, are shown in bold.

	**20 mins**	**40 mins**	**60 mins**
**Control VS BPA**	x	**p=0.005**	**p=0.012**
**Control VS BPF**	x	**p=0.019**	**p=0.007**
**Control VS BPS**	x	**p<0.001**	**p<0.001**
**BPA vs BPF**	x	p=0.717	p=0.736
**BPA vs BPS**	x	p=0.717	p=0.092

Table S8. Statistical differences in the time profile of contraction induced by 5-HT (1 μM), in HUASMC incubated with BPA, BPF and BPS (20 μM) and subjected to a concentration of SNP (10 μM)(A) and Nif (1 μM)(B). The differences considered statistically significant by the one-way ANOVA test, followed by Dunn's post-hoc test (A) and the Holm-Sidak post-hoc test (B) are shown in bold.

	**(A)**<br>**40 mins**	**(B)**<br>**40 mins**
**Control VS BPA**	x	p=0.432
**Control VS BPF**	x	p=0.868
**Control VS BPS**	x	**p=0.013**
**BPA vs BPF**	x	p=0.432
**BPA vs BPS**	x	p=0.088

Table S9. Statistical differences in HUASMC cell viability after the effect of BPA; BPF and BPS (0.0002 - 1000 μM). Statistically significant differences by the one-way ANOVA test, followed by Dunn's post-hoc test, are shown in bold.

	**0.0002 μM**	**0.002 μM**	**0.02 μM**	**0.2 μM**	**2 μM**	**20 μM**	**100 μM**	**200 μM**	**1000 μM**
**Control vs BPA**	p=1.000	p=1.000	p=1.000	p=1.000	p=1.000	p=1.000	p=1.000	p=0.076	**p<0.001**
**Control vs BPF**	p=1.000	p=1.000	p=1.000	p=1.000	p=1.000	p=1.000	p=1.000	**p<0.001**	**p<0.001**
**Control vs BPS**	p=1.000	p=0.894	p=1.000	p=1.000	p=0.269	p=0.814	p=0.134	**p<0.001**	**p<0.001**
**Ethanol 0.2% vs BPA**	p=1.000	p=1.000	p=1.000	p=1.000	p=1.000	p=1.000	p=1.000	p=0.061	**p<0.001**
**Ethanol 0.2% vs BPF**	p=1.000	p=1.000	p=1.000	p=1.000	p=1.000	p=1.000	p=1.000	**p<0.001**	**p<0.001**
**Ethanol 0.2% vs BPS**	p=1.000	p=1.000	p=1.000	p=1.000	p=1.000	p=1.000	p=1.000	**p<0.001**	**p<0.001**
**Ethanol 1% vs BPA**	p=1.000	p=1.000	p=1.000	p=1.000	p=1.000	p=1.000	p=1.000	p=1.000	**p<0.001**
**Ethanol 1% vs BPF**	p=1.000	p=1.000	p=1.000	p=1.000	p=1.000	p=1.000	p=1.000	**p=0.004**	**p<0.001**
**Ethanol 1% vs BPS**	p=1.000	p=1.000	p=1.000	p=1.000	p=1.000	p=1.000	p=1.000	**p=0.002**	**p<0.001**
